# A case report of delayed lower intestinal bleeding after organophosphate poisoning

**DOI:** 10.1186/s12876-021-01981-5

**Published:** 2021-10-29

**Authors:** Wei Hung, Tsung-Heng Tsai, Jian-Han Chen

**Affiliations:** 1 Department of Medicine, E-DA Hospital, Kaohsiung, Taiwan; 2 Department of Pathology, E-DA Hospital, Kaohsiung, Taiwan; 3 Division of General Surgery, Department of Surgery, E-DA Hospital, Kaohsiung, Taiwan

**Keywords:** Organophosphate poisoning, Gastrointestinal bleeding, Intestine ulcer, Case report

## Abstract

**Background:**

Organophosphate poisoning is a serious issue and it results in significant casualties in developing countries. Since agriculture remains an important and necessary sector of human society and organophosphate are commonly used in agriculture, it is difficult to prevent organophosphate poisoning. Gastrointestinal bleeding is not a common but life threatening symptom of organophosphate poisoning. We report a rare case of gastrointestine bleeding due to organophosphate poisoning.

**Case presentation:**

A 78-year-old woman presented to our hospital approximately 12 h after ingesting a mouthful of organophosphate and benzodiazepines in a suicide attempt. Six weeks after successful medical treatment for respiratory failure, she developed recurring melena. Colonoscopy and esophagogastroduodenoscopy findings were negative for ulcers or bleeding. Enteroscopy revealed severe circumferential ulcers with luminal narrowing 10 cm proximal to the ileocecal valve. The patient underwent a 100-cm ileum resection after failed medical treatment and recovered uneventfully. The resected terminal ileum demonstrated severe inflammation and a sharp transitional zone between the healthy and injured mucosa approximately 50 cm proximal to the ileocecal valve. Pathological examination revealed an injured mucosa with inflammatory cell infiltration and structural damage. This case highlights a rare event of OP poisoning with late-onset lower gastrointestinal bleeding, which prolonged the patient’s recovery course and parenteral alimentation period.

**Conclusion:**

We report a rare case of a patient with organophosphate poisoning, with late-onset lower GI tract bleeding, which raised clinical awareness regarding the organophosphate poisoning that induce intestinal symptoms.

## Background

Organophosphate (OP) poisoning is a serious public health issue in developing countries, and it results in significant casualties. Although the actual number of OP poisoning cases is difficult to determine based on surveillance, it is believed that OP poisoning causes approximately 250,000–350,000 deaths each year globally, and studies have reported mortality rates ranging from 3 to 10% [1–3]. Since agriculture remains an important and necessary sector of human society and OPs are commonly used in agriculture, it is difficult to prevent OP poisoning in many countries.

Gastrointestinal (GI) bleeding is not a common symptom of OP poisoning; however, several reports have indicated that this complication may be serious and even life threatening [4, 5]. Although technological improvements have made the detection of GI bleeding more efficient and precise, it still takes days to diagnose and treat the causative factor in most cases. Here, we present an atypical case and a treatment course that could help clinicians recognize the variability of symptoms in OP poisoning and provide patients with better treatment efficiently.

## Case presentation

A 78-year-old woman was brought to our emergency room by her family 12 h after consuming a mouthful of glyphosate-isopropylammonium (organophosphate) and an unknown amount of benzodiazepine. She had suffered from an episode of unconsciousness and vomiting. She had hypertension, hypothyroidism, gastric ulcer, Parkinson's disease, heart disease, and depressive disorder.

On arrival, her Glasgow Coma Scale was E (eye opening)/V (verbal response)/M (motor response): 1/2/4, with her bloodwork revealing severe metabolic acidosis (pH = 7.08; normal pH, 7.35–7.45), 86% peripheral oxygen saturation (SpO_2_), and presence of benzodiazepines (296.5 ng/dl). Chest X-ray revealed bilateral lung infiltrates. She was first intubated and then shifted to the intensive care unit (ICU) owing to progressive dyspnea and deteriorating consciousness. After 41 days of intensive unit care, the patient was transferred to the general ward and recovered uneventfully with tracheostomy and total parenteral nutrition.

We resumed enteral nutrition via nasogastric feeding after she was transferred to the general ward. However, we observed intermittent bloody stool with decreased hemoglobin level after resuming intestinal nutrition. We performed both upper GI endoscopy and colonoscopy but could not identify any possible bleeders. Enteroscopy revealed circumferential ulcers that bled easily on touch with luminal narrowing 10 cm above the ileocecal valve. The ileal biopsy report was negative for cytomegalovirus antigen or tuberculosis. We concluded the bleeding to be intractable because it was not cured even after 2 weeks of conservative treatments, including pharmacological treatment with tranexamic acid and several blood transfusions, the patient’s hemoglobin level was approximately 7–9 g/dl; therefore, we decided to perform surgical intervention.

**Fig. 1:**
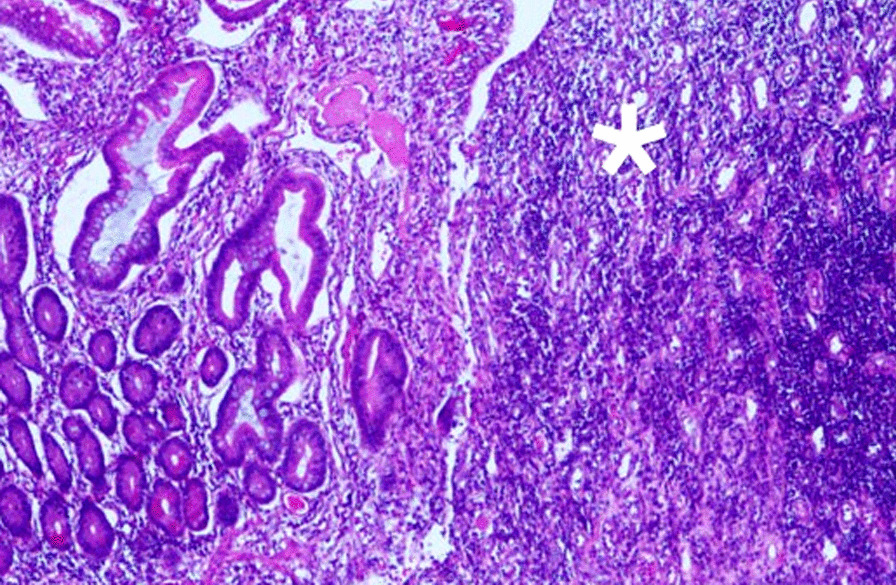
The transitional zone with a sharp and obvious margin. The injured mucosa with inflammation and stenosed tissue is marked

**Fig. 2:**
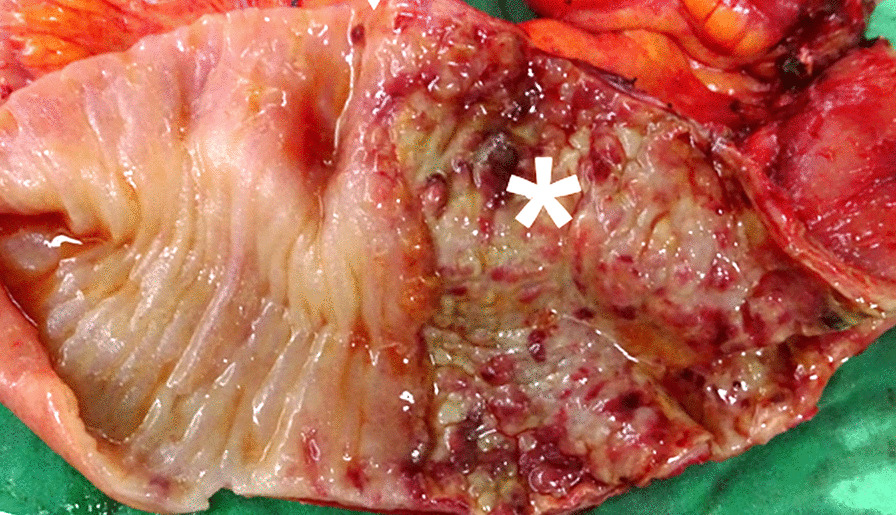
The transitional zone under 40 × 10 magnification with hematoxylin and eosin (HE) stain. The injured mucosa is marked. The right side of the picture denotes the relatively healthy mucosa with the gland tissue still preserved

Exploratory laparotomy revealed severe adhesions over the pelvic space. The terminal ileum exhibited severe inflammation with stenosis 10 cm distal to the ileocecal valve. The transitional zone between the healthy and injured mucosa was approximately 50 cm proximal to the ileocecal valve (Fig. 1). The histopathological report revealed injured mucosa with inflammatory cell infiltration and structural damage. It was composed of neutrophils, plasma cells, and lymphocytic infiltration with vascular granulation tissue that was negative for acid-fast staining (Fig. 2). No other organic lesions or malignancies were noted after meticulously checking the entirety of the small intestine. Finally, we resected 100 cm of the ileum till the healthy bowel and reconstructed it with side-to-side ileocecal anastomosis. Postoperatively, the patient resumed enteral nutrition 5 days after bowel resection and was discharged uneventfully 20 days after the surgery, the 84th day of hospitalization, with tracheostomy and a soft food diet.

## Discussion and conclusions

We report about a 78-year-old woman who drank a mouthful of OP and developed delayed GI bleeding 6 weeks after pesticide consumption. The clinical symptoms of the patient were unique for OP poisoning; this was a rare event of OP poisoning that was followed by late-onset lower GI bleeding, which prolonged the patient’s recovery course and parenteral alimentation period.

OP toxicity is caused by its ability to inhibit acetylcholinesterase, leading to the accumulation of acetylcholine, which causes the overstimulation of muscarinic and nicotinic receptors in the peripheral and central nervous systems [3]. Hence, the symptoms of OP poisoning are related to neural functions and can be divided into three categories: acute (within 24 h), subacute (24 h to 2 weeks; e.g., extra-pyramidal symptoms, miosis, and bradycardia), and chronic (beyond 2 weeks; e.g., peripheral neuropathy) [6].

Acute symptoms, including salivation, lacrimation, urination, defecation, gastric cramps, and emesis (SLUDGE), may occur within minutes to hours and are pathognomonic to the early stages of the disease [6]. The most severe and life-threatening symptom of OP poisoning is respiratory failure, and our case presented with the same. The decrease in respiratory function may have resulted in central neurological apnea [7].

GI symptoms that present in OP poisoning cases mostly include diarrhea, stomach cramps, and increased salivation [6]. Nevertheless, GI bleeding has also been reported in several cases [4, 5, 8]. This can be in the form of upper GI bleeding, delayed colonic bleeding, or multiple small intestinal perforations. In cases of acute small intestinal perforation, patients may succumb to the illness over a span of several days [5]. The treatment and prognosis of OP-induced small intestinal bleeding were difficult to address in our case. To the best of our knowledge, this is the only case presenting a feasible treatment for OP-induced small intestinal bleeding.

The small intestine is an uncommon site of GI bleeding. The common causes of small intestinal bleeding include angiodysplasia, inflammation, and tumors. Products of technological progress such as capsule endoscopy, CT enterography, and deep enteroscopy have improved our ability to investigate this symptom [9]. Fortunately, our patient’s lesion was close to the ileocecal valve and was discovered by enteroscopy.

Intestinal ulcers caused by different factors have been reported in several studies, and their treatments vary according to the etiology of the bleeding. It can be treated by conservative, radiological, pharmacological, endoscopic, and surgical methods [9–11]. A novel and developing treatment of GI bleeding is endoscopy, and the affinity for surgical intervention is gradually declining, with only 18–25% of patients undergoing surgery [12]. However, our patient’s lesion was a segmental erosion ulcer with luminal narrowing and bleeding. Epinephrine injection, thermal or electrical coagulation, or endoscopic clips were not suitable for this circumstance, so surgical intervention was applied.

Since the position of the erosion was at the end of the small intestine, we suspect that there was some intraluminal substance accumulation due to valve closure, which caused the peripheral ulcer formation. The form of the ulcer is uncommonly seen in the intestine of an OP poisoning patient. An old paper which summarizes nine patients with circumferential intestinal ulcers has also mentioned that the cause of this type of ulcer was idiopathic [13]. In some articles, OP-induced internal bleeding has been reported [4, 5, 8]. In two reports, OPs caused GI bleeding, and bowel perforation was considered to have resulted in microvascular thrombosis, which led to bowel wall ischemia. A theory explains the formation of ischemic enteritis, because OP poisoning is a known inducer of vascular endothelial dysfunction and contributes to the formation of small thromboses in small vessels near the point of accumulation, which finally leads to delayed ischemic bowel lesions [4, 5].

A trial with rats, on the other hand, proposed some other reason for the OP-induced inflammatory changes in the bowel [14]. They focused on how the oxidative stress OPs induce in the intestine influences the gene expression of intestinal cells. To assess whether OPs can induce oxidative stress in the small intestine, total superoxide dismutase (T-SOD), malondialdehyde (MDA), H_2_O_2_ (hydrogen peroxide), glutathione(GSH), and glutathione peroxidase (GSH-Px) were measured. The anti-oxidant enzymes T-SOD, GSH, and GSH-Px were significantly decreased (*p* < 0.05), while oxidative stress factors such as malondialdehyde (MDA) and H_2_O_2_ were significantly increased (*p* < 0.05) in the presence of OPs. Then, they analyzed the expression of IL1b, IL-6, TNF-a, MAPK3, NF-kB, and caspase-3 and found that these inflammation- and apoptosis-related mRNA molecules were significantly (*p* < 0.05) increased in OP-treated rat cells. This report indicates that inflammation caused by the cell death process and oxidative stress could be induced by OPs in rats [14].

Although the deposition and absorption of OPs in the body is not entirely clear, there is a case report of secondary ileocecal valve obstruction due to theophylline toxicity with activated charcoal administration, which was managed with surgical intervention [15]. However, obstruction above the ileocecal valve is not common in healthy people. There is a review mentioning 14 heroin couriers, of which two had their solid package in their stomachs, while 12 of them successfully passed the package out of body [16]. In our case, the patient drank a mouthful of OP which was in liquid form. Thus, if solid material can be passed with ease, it seems unlikely that the liquid OP obstructed the ileocecal valve. It is possible that OP intoxication induced the ileocecal valve smooth muscles to constrict, which caused this rare phenomenon.

In conclusion, this case highlights a rare case of a patient with OP poisoning, with late-onset lower GI tract bleeding, which substantially prolonged the patient's recovery course and parenteral alimentation period. Therefore, except for SLUDGE symptoms, attention should be paid to the OPs that induce intestinal symptoms and ensure that the problem is controlled well in time.

## Data Availability

All data generated or analyzed during this study are included in this publish article.

## Abbreviations

OP: Organophosphate
GI: Gastrointestinal
SLUDGE symptoms: Salivation, lacrimation, urination, defecation, gastric cramps, and emesis
T-SOD: Total superoxide dismutase
MDA: Malondialdehyde
H_2_O_2_: Hydrogen peroxide
GSH: Glutathione
GSH-Px: Glutathione peroxidase
MDA: Malondialdehyde
